# Systematic Review of PET/CT Utilization in Breast Implant-Associated Anaplastic Large Cell Lymphoma

**DOI:** 10.3390/medicina61122160

**Published:** 2025-12-04

**Authors:** Mihaela Raluca Mititelu, Teodora Sidonia Mititelu, Dumitru Crăciun, Ștefan Bogdan Solomon, Ciprian Tutui, Andrei Iulian Rugină, Silviu Adrian Marinescu

**Affiliations:** 1 Department of Nuclear Medicine, University of Medicine and Pharmacy “Carol Davila”, 020021 Bucharest, Romania; raluca.mititelu@umfcd.ro (M.R.M.);; 2 Clinic of Nuclear Medicine, University Emergency Central Military Hospital “Dr. Carol Davila”, 024185 Bucharest, Romania; 3 Department of Plastic and Reconstructive Surgery, University of Medicine and Pharmacy “Carol Davila”, Blvd. Eroii Sanitari Nr. 8, Sector 5, 050474 Bucharest, Romania; andrei-iulian.rugina@drd.umfcd.ro (A.I.R.); silviu.marinescu@umfcd.ro (S.A.M.); 4 Department of Plastic and Reconstructive Surgery, “Bagdasar-Arseni” Clinical Emergency Hospital, 041915 Bucharest, Romania

**Keywords:** breast implant-associated anaplastic large cell lymphoma, PET/CT, BIA-ALCL

## Abstract

*Background and Objectives*: Breast Implant-Associated Anaplastic Large Cell Lymphoma (BIA-ALCL) is a T-cell lymphoma that has shown an interest in the medical community in recent years. Given its emerging clinical relevance, accurate imaging plays a crucial role in diagnosis, staging, and follow-up. This systematic review aims to evaluate the role of Fluorine-18 Fluorodeoxyglucose positron emission tomography/computed tomography (18F-FDG PET/CT) in the staging and follow-up of patients with BIA-ALCL, focusing on its diagnostic accuracy and clinical impact. *Materials and Methods*: A systematic search of PubMed and National Center for Biotechnology Information (NCBI) was conducted to identify studies assessing the use of 18F-FDG PET/CT in BIA-ALCL up to and including 15 April 2024, using the following keywords “breast implant-associated anaplastic large cell lymphoma” AND “PET/CT” AND “BIA-ALCL”. Data regarding the role of PET/CT in disease detection, staging, therapeutic guidance, and post-treatment surveillance was analyzed and synthesized in a tabulated format for comparative analysis. Given study heterogeneity, findings were synthesized narratively and diagnostic performance metrics were summarized descriptively, and no formal risk-of-bias assessment was performed due to the descriptive, case-based nature of evidence. *Results*: A total of 28 studies met the inclusion criteria, comprising 27 individual case reports and one case series that included seven patients. Across these studies, 18F-FDG PET/CT demonstrated diagnostic utility in the evaluation of BIA-ALCL, serving primarily for initial disease staging in 27 cases and for monitoring treatment response in 16 cases. Discussion: The review’s limitations include potential search bias due to variable radiotracer terminology and the restriction to English-language studies, which may limit literature retrieval and generalizability. *Conclusions:* Thus, 18F-FDG PET/CT demonstrated significant value in early lesion detection, accurate staging, assisting in monitoring treatment response and detecting recurrence.

## 1. Introduction

Breast implants are widely used in both aesthetic augmentation and reconstructive procedures across various clinical contexts. According to the 2024 report by the American Society of Plastic Surgeons, breast augmentation continues to represent one of the most frequently performed aesthetic surgeries. With a total of 306,196 procedures reported in 2024, it ranked as the second most common aesthetic intervention, reflecting a 1% increase compared to 2023. Breast reconstruction has remained among the five most frequently performed reconstructive procedures for the third consecutive year, with a 3% increase in the number of patients compared to the previous year. It is noteworthy that the rate of breast implant removals among reconstructive patients has also increased by 5% [1].

There are two main types of breast implants: saline- and silicone gel-filled, both consisting of a silicone elastomer shell with either a smooth or textured surface. Although the use of silicone implants was suspended in the United States between 1992 and 2006, they have since become the predominant choice in breast surgery, whereas saline implants are now considered a secondary option due to higher rates of deflation and volume inconsistencies related to under- or overfilling [2]. Regarding surface characteristics, textured implants adhere to the surrounding fibrous capsule, aiding in the maintenance of implant position and orientation, particularly in anatomically shaped ones. The first textured implant was introduced in 1968, and its use expanded considerably during the 1990s, as it was believed to reduce the incidence of capsular contracture. It is currently estimated that more than 35 million textured breast implants have been placed worldwide [3].

Although implant rupture and capsular contracture remain the leading causes of implant failure, women with breast implants may also experience delayed-onset breast swelling, which is benign in most cases. However, there are emerging instances of breast malignancies, clinically similar, presenting with delayed seromas, such as: breast implant-associated anaplastic large cell lymphoma (BIA-ALCL), breast implant-associated diffuse large B-cell lymphoma (BIA-DLBCL), and breast implant-associated squamous cell carcinoma (BIA-SCC) [4]. Therefore, this systematic review aims to centralize and synthesize all published case reports and case series of BIA-ALCL to summarize different patient presentations, patterns of disease extension, diagnostic approaches, management strategies, and outcomes.

The first case of BIA-ALCL was described in 1997 by Keech and Creech in a patient with recurrent treatment-resistant breast implant effusion. The scale of this condition gained significant attention and now is part of routine informed consent for breast implants. As a result, some textured implant subtypes are now prohibited in various countries [5]. As of 30 June 2024, the U.S. FDA has received a total of 1380 U.S. and global medical device reports of BIA-ALCL [6].

BIA-ALCL most commonly presents with rapid and noticeable swelling due to periprosthetic effusion (85%) or, less frequently, as a palpable mass adjacent to the implant (15%) in the affected breast. Pain is reported in roughly one-third of patients, while approximately one-fourth present with cutaneous manifestations, primarily skin erythema, subcutaneous nodules, eruptions, erosions, or ulcers [5]. In rare instances, systemic “B symptoms” such as fever, lymphadenopathy, night sweats, and fatigue may occur. The disease typically spreads locally, mainly to the ipsilateral axillary nodules with less frequent involvement of mediastinal, supraclavicular or internal mammary nodules. In rare cases, distant spread to the bone marrow or distant lymph nodes has been reported [1].

Breast ultrasound is considered the imaging modality of choice for the evaluation of periprosthetic fluid collections, regional lymphadenopathy, or peri-implant masses. Any detected effusion should be aspirated and submitted for cytologic examination. In cases where cytologic findings are inconclusive, breast MRI may be considered, as it offers high sensitivity for detecting and characterizing periprosthetic effusions. When a mass is identified, tissue biopsy is essential to achieve definitive histopathologic characterization and diagnosis [7].

Nuclear medicine imaging, particularly 18F-FDG PET/CT, is a standardized and well-established modality for evaluating hematologic malignancies. The key clinical advantages of PET/CT include whole-body coverage, accurate lesion characterization, comprehensive disease staging, and early assessment of treatment response. This technique is especially valuable in patients with high-grade lymphomas such as Hodgkin lymphoma and NH-DLBCL [5]. Although most cases of BIA-ALCL present at an early stage, 18F-FDG PET/CT is generally recommended as the initial imaging modality for complete disease staging. According to the NCCN guidelines (Version 2.2025), 18-FDG PET/CT is preferred over CT preoperatively due to the possibility of extranodal disease, which may be inadequately imaged by CT alone [8].

Patients with Stage I BIA-ALCL typically present with a periprosthetic effusion, early capsular invasion, or a mass confined within the fibrous capsule. Although extracapsular spread is identified in over 25% of cases, many of these are still categorized as Stage I due to limited nodal or systemic involvement [4]. In Stage IIA or IIB disease, there is often tumor extension beyond the capsule and/or involvement of a single regional lymph node. Stage III is characterized by localized lymph node involvement with extracapsular tumor extension [9]. When dissemination extends beyond the ipsilateral breast and regional lymph nodes, the disease is classified as Stage IV. In the literature, advanced cases have been reported with secondary involvement of the bones, central nervous system, liver, and small intestine (Table 1).

18F-FDG PET/CT plays a pivotal role in the initial staging and detection of distant disease, particularly in mass-forming BIA-ALCL [5]. It is essential that PET/CT be performed prior to surgery, as postoperative inflammatory changes may cause false-positive results. Baseline imaging also provides a reference point for subsequent comparative assessments during follow-up or after different treatment modalities.

Surgical excision remains the first-line therapy for early-stage BIA-ALCL, with complete capsulectomy and implant removal often achieving curative outcomes. In patients with advanced disease involving the thoracic wall or lymph nodes, systemic therapy is indicated. Immunotherapy targeting CD30-positive cells—brentuximab vedotin, either alone or combined with traditional chemotherapy regimens such as CHOP (cyclophosphamide, doxorubicin, vincristine, and prednisolone), as well as adjuvant radiotherapy, has demonstrated favorable outcomes. Removal of the contralateral implant is also advised, and reconstruction using autologous tissue is preferred, given the absence of conclusive evidence excluding other implant types from potential risk [4,9,10].

To confirm disease remission, both clinical and paraclinical evaluations should be conducted at regular intervals. Follow-up typically incorporates multimodal imaging, with PET/CT considered the most accurate modality for assessing metabolic response (particularly when a pre-treatment scan is available for comparison). Patients without a solid mass who were monitored every 3–6 months for two years remained disease-free, whereas those presenting with a mass exhibited a worse prognosis. Overall, the median projected survival is approximately 12 years, with reported survival rates of 97% at 3 years and 92% at 5 years. However, when disease extends beyond the capsule, the 5-year survival decreases to approximately 72%, especially in cases with a mass-forming presentation, which represents an adverse prognostic indicator [11].

## 2. Materials and Methods

A comprehensive literature search was conducted using free and open-access centralized databases, including PubMed and the NCBI Central Repository. The search strategy employed the following keywords: “breast implant-associated anaplastic large cell lymphoma” AND “PET/CT” AND “BIA-ALCL”. The search encompassed all available literature up to 15 April 2024, which was the date of the last consultation for both databases. The primary outcomes of interest were the diagnostic utility of 18F-FDG PET/CT for early detection, accurate staging, and follow-up assessment of BIA-ALCL. Reviews were excluded if they consisted solely of abstracts, did not focus on BIA-ALCL, did not utilize PET/CT imaging, or represented secondary research such as reviews, systematic reviews, or clinical guidelines.

**Figure 1:**
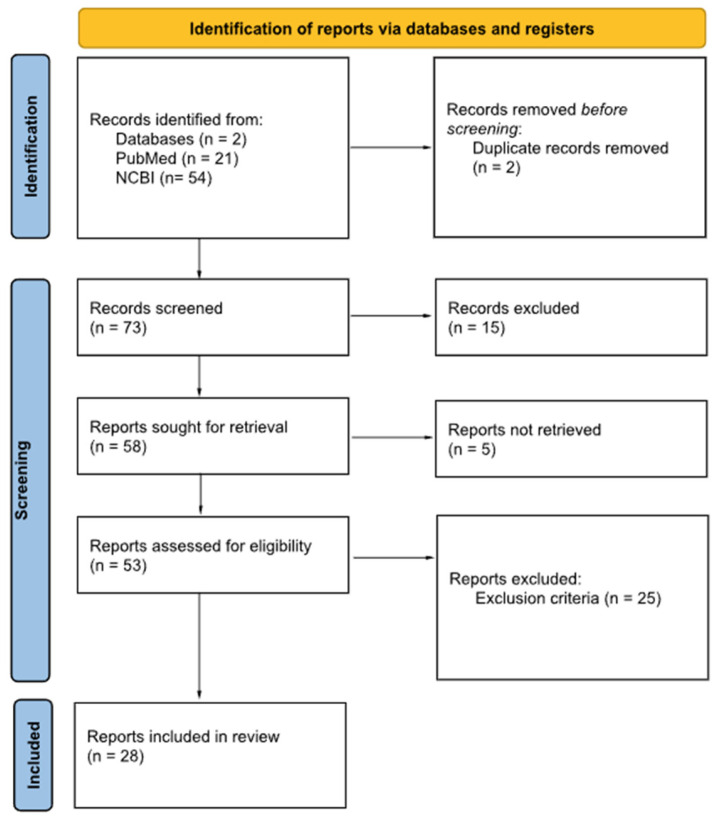
Selection procedure.

The study selection process for this systematic review involved a comprehensive multi-stage screening procedure. An initial search identified 75 articles related to BIA-ALCL and PET/CT across the selected databases with two duplicate records being removed. Of these, 15 records were excluded for not meeting the predefined inclusion criteria, and five were removed because only abstracts were available. An additional 25 articles were omitted as they represented secondary research (including reviews, systematic reviews, or guidelines). Following the application of all inclusion and exclusion criteria, a total of 28 articles were retained for qualitative analysis and summarized in Figure 1.

For objective and reliable results during the review process, three reviewers independently screened each article to determine eligibility based on the inclusion criteria. The results of the screening process were centralized in a spreadsheet format with no noticeable discordance between the reviewers. The data extracted from each study included bibliographic details (author, year, title, DOI, PubMed Central ID, publication date, and study link), study characteristics (study type and number of patients), and clinical variables relevant to PET/CT application in BIA-ALCL, including staging, disease extension, follow-up findings, and additional information related to autoimmune involvement or other notable observations.

This systematic review was qualitative in nature and therefore, no statistical calculation or quantitative synthesis was performed. Reported outcomes such as sensitivity, specificity, and descriptive diagnostic performance metrics were extracted when available; no quantitative synthesis was conducted. All the findings from the included studies were summarized in tabular form. Given the descriptive scope and heterogeneity of the included reports, formal risk-of-bias assessment was not performed. Reporting bias and certainty of evidence were not formally assessed due to the qualitative nature of the review.

This systematic review was conducted and reported in accordance with the PRISMA 2020 guidelines. This review protocol was entered into the PROSPERO (CRD420251235304). No data, code, or materials have been deposited in a public repository; all extracted data are provided within the manuscript.

## 3. Results

On average, most case reports within the dataset featured one patient, with the largest reported count of seven cases per report (Table 2). Among all case reports (n = 28), 27 described the use of 18F-FDG PET/CT for initial disease staging, while 16 additionally reported follow-up PET/CT examinations. Most patients demonstrated favorable outcomes following surgical management, with PET/CT scans confirming the absence of metabolic activity consistent with residual or recurrent disease. In a limited number of reports, PET/CT also detected extranodal involvement in two patients, underscoring its diagnostic value in comprehensive disease assessment. No statistical syntheses or meta-analyses were performed.

## 4. Discussion

Lymphomas are hematologic malignancies broadly classified into Hodgkin and non-Hodgkin lymphomas (NHL). The latter encompasses a diverse group of subtypes, each characterized by distinct epidemiological, etiological, genetic, and clinical features, as well as various responses to therapy. Non-Hodgkin lymphomas are further categorized as either “indolent” or “aggressive,” based on their biological behavior and prognosis. Peripheral T-cell lymphomas (PTCL) constitute a heterogeneous group of mature T-cell and natural killer (NK) cell neoplasms, accounting for less than 10% of all NHL cases. Within this group, Anaplastic Large Cell Lymphoma (ALCL) is a distinct subtype characterized by CD30-positive anaplastic cells. Which is further divided into two major subtypes based on the expression of the anaplastic lymphoma kinase (ALK) protein: ALK-positive and ALK-negative Large Cell Cutaneous Ki-1 Anaplastic Lymphoma. The current WHO classification identifies approximately 30 PTCL entities, categorized according to clinical presentation as predominantly leukemic, nodal, extranodal, or cutaneous diseases.

ALCL accounts for approximately 2% of all NHL cases and represents the third most common subtype of adult nodal T-cell lymphomas, following peripheral T-cell lymphoma not otherwise specified (PTCL-NOS) and angioimmunoblastic T-cell lymphoma [7]. It is important to note that ALCL shares several defining features, including large-cell anaplastic morphology, robust CD30 expression and frequent activation of phospho-STAT3. BIA-ALCL is recognized as a distinct clinicopathological subtype within the spectrum of ALK-negative ALCL [39].

The etiology of BIA-ALCL remains incompletely understood; however, evidence suggests a strong association with the use of textured breast implants, with onset typically occurring more than one year after implantation and a median of 8–10 years. Multiple articles have correlated specific implant surface characteristics and the development of BIA-ALCL. Notably, the risk of BIA-ALCL has been reported significantly higher among patients with Silimed polyurethane implants—approximately 23.4 times greater than with Biocell implants and 16.5 times greater than with Siltex implants. These findings highlight a potential association between implant surface area/roughness and a higher BIA-ALCL incidence [40].

One of the leading hypotheses regarding the pathogenesis of BIA-ALCL proposes that Gram-negative bacteria colonize the biofilm on the surface of textured implants, leading to lymphocyte activation. Progressive tissue integration into the textured implant surface may further stimulate innate immune pathways, promoting T-cell proliferation and maintaining a state of chronic inflammation [40]. This persistent inflammatory environment is thought to contribute to malignant transformation of T cells, typically exhibiting an ALK-negative and CD30-positive immunophenotype [3]. Viral involvement has also been proposed, as suggested by rare reports of Epstein–Barr virus (EBV)-positive diffuse large B-cell lymphoma occurring in association with breast implants and demonstrating CD30 expression. These findings underscore the need for continued investigation into the mechanisms of implant-associated lymphomagenesis to ensure accurate differentiation from BIA-ALCL [31].

Genetic predisposition is also considered an important risk factor in the development of BIA-ALCL. In a study employing whole-exome sequencing of DNA extracted from cytologic fluid and germline samples from two patients with effusion-limited BIA-ALCL, mutations in JAK1 and STAT3, as well as a germline JAK3 mutation, were identified. The presence of the germline JAK3 variant suggests a potential inherited susceptibility contributing to lymphomagenesis in this disease [41]. Furthermore, specific HLA polymorphisms may increase individual predisposition to BIA-ALCL. Characterizing variations in HLA alleles among affected patients could help identify individuals with textured implants who are at relatively higher or lower risk of developing the disease over their lifetime [40]. In addition, a chronic IgE-mediated hypersensitivity response has been proposed as another possible contributing factor, supported by the detection of elevated inflammatory cytokines such as IL-6 and IL-1 in the periprosthetic environment [42].

Several reports in the literature have described an association between ALCL and various types of implants, including dental, gluteal or silicone-based breast implants. It has been proposed that chronic mechanical stimulation or frictional forces exerted by these implants on surrounding tissues may contribute to local inflammation. Experimental animal models lend support to this hypothesis, demonstrating comparable periprosthetic inflammatory responses. However, definitive evidence confirming a direct pathogenic role of silicone implants in this process remains lacking [9]. Overall, the pathogenesis of BIA-ALCL is likely multifactorial, involving a complex interplay between host-related factors, such as immune dysregulation or genetic susceptibility, and environmental influences.

When evaluating diagnostic imaging modalities, ultrasound demonstrated the highest sensitivity (84%) for detecting periprosthetic effusions, followed by MRI (82%), chest CT (55%), and PET/CT (38%). In contrast, for mass detection, the sensitivity rates were 46%, 50%, 50%, and 64% for ultrasound, chest CT, MRI, and PET/CT, respectively [43]. Given that PET/CT shows superior sensitivity for identifying masses, whereas ultrasound excels in detecting effusions, a multimodal imaging approach is recommended to optimize diagnostic accuracy. A key limitation of 18F-FDG PET/CT lies in its reliance on glucose metabolism, which prevents differentiation between benign and malignant peri-implant effusions and may lead to false-negative interpretations [30].

Conversely, false-positive results may occur on initial PET/CT due to inflammatory or infectious processes, potentially resulting in overestimated TNM staging. Similarly, during post-therapeutic evaluation, inflammatory lymph node reactions following surgery or treatment can produce false-positive findings. To minimize such confounding effects, adherence to optimal timing for post-treatment PET/CT imaging is essential. Current recommendations suggest performing scans at least 2–3 months after radiotherapy, 2–3 weeks after chemotherapy, and 6 weeks after surgery. Failure to comply with these intervals may compromise diagnostic accuracy and the clinical utility of PET/CT in BIA-ALCL management, emphasizing the importance of integrating imaging results with surgical and clinical context [14,24]. Based on the NCCN Guidelines 18F-FDG PET/CT is not recommended for routine screening in asymptomatic patients. Instead, initial evaluation of symptomatic typically favors ultrasound of MRI. However, the use of PET/CT becomes essential once BIA-ALCL is suspected or histologically confirmed. This is preferred since it accurately stages the disease and detects extranodal involvement, which is a common characteristic of T-cell lymphomas, surpassing CT imaging alone. Following surgical treatment that achieves complete excision and no residual disease, 18F-FDG PET/CT may be considered for disease surveillance, typically performed no more frequent than every six months for the first two years [13].

In cases where early diagnosis and prompt surgical intervention were achieved, postoperative PET/CT scans consistently demonstrated favorable outcomes, with no residual foci of 18F-FDG uptake. This further supports the role of PET/CT in comprehensive disease surveillance and confirmation of complete remission [17,22,24,25,27,34,35].

The use of 18F-FDG PET/CT provides several advantages in evaluating therapeutic response. By detecting metabolic alterations preceding structural changes, PET/CT enables early assessment of treatment efficacy and facilitates response-adapted therapy. The standardized uptake value (SUV), particularly the maximum SUV (SUVmax), provides a semiquantitative measure of metabolic activity and allows comparison of pre- and post-treatment findings. This approach aids in evaluating treatment response whether complete, partial, stable, or progressive and guides therapeutic decision-making [44,45].

An additional population of clinical relevance includes male-to-female transgender patients, of whom approximately 60–70% undergo breast augmentation procedures in order to achieving feminization of the chest. With the increasing number of patients referring to gender identity clinics for gender dysphoria, clinicians should remain vigilant regarding the potential risk of BIA-ALCL following surgery. Unlike breast cancer patients enrolled in structured surveillance programs, transgender or aesthetic augmentation patients are less likely to undergo routine postoperative follow-up, posing a risk of delayed diagnosis [12,20,21,36,38].

Incorporating case reports into a systematic review presents inherent challenges, including selection bias toward rare or unusual presentations, limited generalizability, publication bias favoring positive outcomes, and heterogeneity in reporting standards. The lack of methodological rigor and standardized data acquisition further complicates synthesis and comparison. Establishing a systematic and transparent workflow is therefore essential to accurately evaluate the role of 18F-FDG PET/CT in BIA-ALCL, ensuring objectivity and reproducibility across datasets. Moreover, concomitant comorbidities, such as cardiovascular or systemic diseases, can significantly influence clinical management and outcomes [13].

During database searches, the use of the keywords “PET/CT AND breast implant-associated anaplastic large cell lymphoma (BIA-ALCL)” yielded more results compared to queries including “18F-FDG,” despite the latter offering greater radiotracer specificity. This discrepancy likely reflects the widespread clinical adoption of 18F-FDG per guideline recommendations, whereas alternative radiotracers remain under investigation. The assumption that all PET/CT scans utilized 18F-FDG introduces ambiguity, particularly given ongoing research into novel radiotracers. Failure to specify the radiotracer employed may hinder reproducibility and interpretation of findings. Furthermore, search algorithms may prioritize volume over specificity, potentially underrepresenting publications explicitly referencing “18F-FDG.” Consequently, the literature may lack comprehensive coverage of relevant investigations, underscoring the importance of precise terminology in future research on PET/CT imaging in BIA-ALCL [5]. In addition, the search criteria were restricted to English-language literature, thereby limiting the diversity of the patient population represented in the analysis.

## 5. Conclusions

The findings emphasize the role of 18F-FDG PET/CT in the diagnosis, staging, assessment of treatment response, and follow-up surveillance of breast implant-associated anaplastic large cell lymphoma (BIA-ALCL). However, it is essential to acknowledge that while PET/CT offers significant diagnostic and prognostic value, it should be employed in conjunction with complementary imaging and clinical modalities to ensure optimal patient management. Continued multidisciplinary efforts and methodological standardization will enhance understanding of BIA-ALCL and contribute to improving diagnostic precision, therapeutic strategies, and long-term patient outcomes.

## Figures and Tables

**Table 1:** TNM classification of BIA-ALCL (TNM—Tumor, Node, Metastasis; BIA-ALCL—Breast Implant-Associated Anaplastic Large Cell Lymphoma) [10].

Description of Lymphoma Cells		Stage
T—Tumor Extent (penetration of capsule)		
T1	Only in the effusion or on the luminal side of the capsule	1A → T1N0M0
T2	Superficial infiltration of the luminal side of the capsule	1B → T2N0M0
T3	Cell aggregates or sheets penetrate the capsule	1C → T3N0M0
T4	Cells infiltrate beyond the capsule	2A → T4N0M0
N—Nodal Involvement		
N0	No lymph node involvement	
N1	One local or regional lymph node involved	2B → T1–3N1M0
N2	More than one local or regional lymph node involved	3 → T4N1–2M0
M—Metastatic Disease		
M0	No distant metastasis	
M1	Distant metastasis present	4 → T1–4N0–2M1

**Table 2:** Summary of published case reports describing the use of 18F-FDG PET/CT in breast implant-associated anaplastic large cell lymphoma (BIA-ALCL), including staging, follow-up, number of patients, disease extent, and clinical outcomes.

Additional Insights	Follow-Up	Disease Extension	Staging	Number of Patients	Age and Gender	Author, Year
N/A	Yes	Hypermetabolic lesions in the right breast and right axilla, mild uptake in the bone marrow	Yes	1	44 F	Galván, 2023 [11]
The patient was a male-to-female transgender	No	No focal periprosthetic, locoregional, or distant pathological uptake (cT1N0M0)	Yes	1	27 F	Materazzo, 2022 [12]
Died due to cardiovascular complications	Yes	Locally advanced disease, involving the 5th, 6th and 7th left ribs (pT4N0M0)	Yes	1	Not mentioned F	Caputo, 2021 [13]
Adverse effect to chemotherapy which led to uptake in the thymus due to rebound hyperplasia	Yes	Uptake in the breast nodules, enlarged axillary lymph nodes and a mediastinal lesion	Yes	1	40 F	Lazaro-Garcia, 2021 [14]
After the PET/CT scan, a PET/MRI scan was performed	No	Small volume effusion surrounding the left breast implant with mild tracer uptake	Yes	1	55 F	Verde, 2020 [15]
3 out of 4 patients did PET/CT scans to assess disease extension, and the remaining patient did a PET/CT scan postoperatively	Yes	P1: Faint capsular uptake on axial fused PET/CT and maximal intensity projection images	Yes	4	58 F, 56 F, 67 F, 66 F	Pandika, 2020 [16]
		P2: 3 foci of peri-implant uptake and an FDG-avid supraclavicular lymph node, bilateral peri-implant fluid with focal uptake within the left one				
		P3: FDG-avid lymphadenopathy and soft tissues masses in the left breast, axilla, subpectoral and supraclavicular regions;				
N/A	No	P1: Peri-implant uptake with increased uptake in the left axillary lymph node	Yes	2	60 F	Siminiak, 2019 [17]
		P2: Uptake at the lower pole of the left implant and infiltration of lymph nodes in left axillary				
N/A	No	Diffuse uptake in the peri-implant capsule with no metastatic disease	Yes	1	36 F	Montes Fernández, 2019 [18]
N/A	Yes	Multiple lesions in the right breast, two hypermetabolic lymph nodes in the right axilla, a hypermetabolic band posterior to the implant involving the pectoralis minor muscle	Yes	1	58 F	Berlin, 2018 [19]
The patient was a male-to-female transgender	Yes	IE stage on Ann Arbor classification	Yes	1	33 F	Patzelt, 2018 [20]
		T4N0M0				
The patient was a male-to-female transgender	No	IE stage on Ann Arbor classification	Yes	1	56 F	de Boer, 2017 [21]
2 out of 4 patients did a PET/CT scan for staging, the other 2 having done a PET/CT scan after the operation	Yes	P1: Peri-implant uptake, with no axillary or distant uptake	Yes	4	64 F, 57 F, 48 F, 74 F	Shoham, 2024 [22]
		P2: Weak absorption of a lymph node in the left axilla				
N/A	Yes	P1: No evidence of lymphadenopathy or suspicion for lymphoma outside of the left breast	Yes	2	59 F	Mehta-Shah, 2018 [23]
		P2: Hypermetabolic 2-cm mass on the capsule of the left breast implant, 2.5 to 3-cm hypermetabolic lymph nodes in the left axilla				
Out of 7 patients, 3 of them did not do a PET/CT scan	Yes	P1: Uptake in the breast and axillary nodes	Yes	7	44 F, 50 F, 30 F, 59 F, 34 F, 46 F, 64 F	Pluta, 2020 [24]
The authors report that in 2 patients that did preoperative scans, PET/CT overestimated the staging		P2: Not done				
One patient had a false positive result in the post-operative PET/CT scan		P3: Negative				
		P4: Not done				
		P5: Uptake in the breast and axillary nodes				
		P6: Uptake in the breast				
		P7: Not done				
One patient did only a postoperative PET/CT scan;	Yes	P1: Large mixed-density mass with intense FDG activity, deep within and invading the right breast and pectoralis muscles; metastatic disease spread to the lung and bone	Yes	3	48 F, 64 F, 33 F	Chacko, 2018 [25]
		P2: Flattened rim of soft tissue, located inferomedially in the left breast, with ill-defined margins and moderate FDG uptake				
		P3: not done				
The patient was a male-to-female transgender	Yes	4 abnormal hypermetabolic soft tissue densities surrounding the right breast implant	Yes	1	58 F	Ali, 2019 [26]
N/A	Yes	N/A	No	2	58 F, 47 F	Crèvecoeur, 2019 [27]
N/A	N/A	Increased uptake along the anterior chest wall, slightly greater on the right than the left	Yes	1	65 F,	Ezekwudo, 2017 [28]
The patient was a pregnant woman;	Yes	Uptake in the left breast with no capsular mass, nor lymphatic or visceral involvement	Yes	1	40 F	Elia, 2021 [29]
The patient was not evaluated according with the NCCN guideline and BIA-ALCL was initially misdiagnosed	N/A	>3 hypermetabolic lymph nodes along the course of the distal right external iliac vessels	Yes	1	70 F	Maglic, 2021 [30]
N/A	Yes	P1: large tumoral mass at the upper-external quadrant of the right breast, in close contact with the implant with a layer of fluid surrounding the implant	Yes	2	75 F, 45 F	Vets, 2023 [31]
		P2: hypermetabolic lesion in the left breast with moderate uptake in the axillary lymph nodes				
N/A	N/A	Left breast hypermetabolic mass and hypermetabolic but non-enlarged left axillary lymph nodes	Yes	1	85 F	Corines, 2021 [32]
N/A	N/A	Mild radiotracer uptake along the right chest wall mass, moderately intense uptake along the left lower outer breast quadrant and at the mediastinal and hilar nodes, and intense uptake in the left axillary nodes;	Yes	1	75 F	Shepard, 2020 [33]
N/A	N/A	P1: was done postoperative and did not reveal suspicious metabolic activity	Yes	2	42 F, 30 F	de Paule, 2023 [34]
		P2: no areas of increased metabolic activity				
N/A	N/A	The PET/CT scan was done postoperative and was negative for metastatic disease	Yes	1	60 F	Keith, 2017 [35]
N/A	Yes	Small hypermetabolic soft tissue focus anterior to the right breast implant and additional focus of hyperactivity in the colon	Yes	1	55 F	Richardson, 2017 [36]
N/A	N/A	Hypermetabolic activity in the anterior outer quadrant of the breast	Yes	1	59 F	Kim, 2015 [37]
The patient was a male-to-female transgender	Yes	Uptake in the left breast	Yes	1	78 F	Orofino, 2016 [38]
The case presented itself initially with mild leukocytosis with hypereosinophilia						

## Data Availability

No new data were created or analyzed in this study.

## Abbreviations

The following abbreviations are used in this manuscript:

BIA-ALCL	Breast Implant-Associated Anaplastic Large Cell Lymphoma
PET/CT	positron emission tomography/computed tomography
18F-FDG	Fluorine-18 Fluorodeoxyglucose
BIA-DLBCL	breast implant-associated diffuse large B-cell lymphoma
BIA-SCC	breast implant-associated squamous cell carcinoma
FDA	U.S. Food and Drug Administration
NHL	non-Hodgkin lymphomas
PTCL	Peripheral T-cell lymphomas
NK	natural killer cell
ALK	the anaplastic lymphoma kinase
WHO	World Health Organization
PTCL-NOS	peripheral T-cell lymphoma not otherwise specified
MRI	magnetic resonance imaging
NCCN	National Comprehensive Cancer Network
CHOP	cyclophosphamide, doxorubicin, vincristine, and prednisolone
